# Combined procedures with unicompartmental knee arthroplasty: High risk of stiffness but promising concept in selected indications

**DOI:** 10.1051/sicotj/2022002

**Published:** 2022-02-22

**Authors:** Vianney Derreveaux, Axel Schmidt, Jobe Shatrov, Elliot Sappey-Marinier, Cécile Batailler, Elvire Servien, Sébastien Lustig

**Affiliations:** 1 FIFA Medical Center of Excellence, Orthopaedics Surgery and Sports Medicine Department, Croix-Rousse Hospital, Hospices Civils de Lyon 103 Grande rue de la Croix Rousse 69004 Lyon France; 2 Sydney Orthopaedic Research Institute Chatswood Sydney 2067 Australia; 3 University of Notre Dame Australia Orthopaedic Research Institute Sydney 2007 Australia; 4 Hornsby and Ku-Ring Hospital Sydney 2077 Australia; 5 Univ Lyon, Claude Bernard Lyon 1 University, IFSTTAR, LBMC UMR_T9406 25 Avenue François Mitterand 69500 Lyon France; 6 LIBM – EA 7424, Interuniversity Laboratory of Biology of Mobility, Claude Bernard Lyon 1 University 43 boulevard du 11 Novembre 69622 Villeurbanne France

**Keywords:** Knee osteoarthritis, Unicompartmental knee arthroplasty, Bicompartmental knee arthroplasty, Patellofemoral joint arthroplasty

## Abstract

*Introduction*: Unicompartmental knee arthroplasty (UKA) has traditionally been contraindicated in the presence of an ACL deficient knee, bi-compartmental disease, or significant coronal deformity due to concerns regarding increased risk of persisted pain, knee instability, tibial loosening, or progression of osteoarthritis. The aim of this study was to evaluate the outcomes of patients undergoing UKA with an associated surgical procedure in these specific indications. *Method*: This was a retrospective cohort study of patients undergoing UKA between December 2015 and October 2020. Patients were categorized into groups based on associated procedures: UKA + ACL, UKA + HTO, and bicompartmental arthroplasty. Outcomes were assessed using the Knee Society Score (KSS) knee and function scores and the Forgotten Joint Score. Radiological and complication analysis was performed at the last clinical follow-up. *Results*: Thirty-two patients (13 men and 19 women) were included. The mean age was 56.2 years ± 11.1 (range, 33–84) with a mean follow-up of 26.3 months ± 15 (7.3–61.1). There was a significant improvement between the pre-and postoperative KSS Knee (+34.3 ± 16.5 [12–69]), Function (+34.3 ± 18.6 [0–75]), and Total scores (+68.5 ± 29.4 [24–129]) (*p* = 0.001). Seven patients (21.8%) required an arthroscopic arthrolysis for persistent stiffness. Two patients (UKA + PFA and UKA + ACL) underwent revision to TKA. Patient satisfaction was 90%, and mean flexion at last follow-up was 122° ± 6 (120–140). The implant survival rate was 94%. *Discussion*: This study found performing UKA with an additional procedure to address relative contraindications to the arthroplasty in physically active patients with monocompartmental knee arthritis is an efficient strategy with good results at short-term follow-up. It should be reserved for patients where TKA is likely to have unsatisfactory results, and the patient has been fully counseled regarding the management options. Even if there is a high rate of complications with stiffness requiring a re-intervention, the final results are very satisfying with no impact of the reintervention on the clinical result in the short term.

## Introduction

Management active patient with end-stage medial osteoarthritis (OA) presents a treatment challenge for orthopedic surgeons. Total knee arthroplasty (TKA) in this population group often fails to meet expectations or functional demands [1]. Unicompartmental knee arthroplasty (UKA) offers a good solution for higher demand patients motivated to return to physically demanding activities such as sport. Recent studies have highlighted the functional benefits of rapid recovery [2], more physiologic knee kinematics, and better gait parameters than TKA [3, 4].

UKA has traditionally been contraindicated in the presence of an ACL deficient knee, bi-compartmental disease, or significant coronal deformity due to observations of increased risk of tibial loosening, residual pain, knee instability, or progression of the disease [5]. This has led to some studies exploring combined procedure’s with UKA. Replacement of additional compartments (bi-compartmental knee arthroplasty) has been successfully performed in small case series [6]. The possibility to perform UKA with associated procedures to treat an “à la carte” of knee deficiencies, including ACL reconstruction, osteotomy for bony extra-articular deformity, and bicompartmental arthroplasty, appears enticing, however, concerns rightly remain regarding implant survival, complications, and patients reported outcomes (PROMs).

This study aimed to evaluate outcomes of patients undergoing UKA with an associated surgical procedure at minimum 6 months follow-up. The authors hypothesised that performing UKA with associated procedures is an efficient and safe approach for the appropriately selected patient.

## Method

### Patients

**Figure 1:**
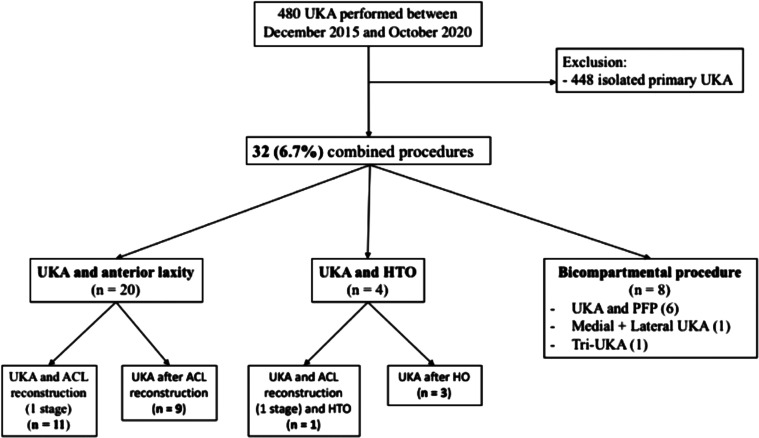
Study flowchart. UKA: unicompartmental knee arthroplasty; HTO: high tibial osteotomy; PFP: patellofemoral prosthesis; ACL: anterior cruciate ligament.

This was a retrospective cohort study of patients undergoing UKA between December 2015 and October 2020 at a single institution. A total of 480 patients underwent UKA in the study period. A flowchart representing patient selection is presented in Figure 1.

Inclusion criteria were UKA performed with an associated ACL reconstruction, osteotomy, patellofemoral prosthesis, bi-UKA, tri-UKA. Exclusion criteria were single compartment, inflammatory arthritis, or primary UKA.

Patient selection for associated procedures was based on the following criteria: Physically active patients with a goal of a return to sporting activities after who suffered from localized knee OA. In all cases, patients had presented following previous knee surgery (meniscectomy, ACL reconstruction, HTO, unicompartmental prosthesis) or with an associated lesion (ALC tear, extra-articular deformity) and had been offered TKA. In all cases, patients had refused TKA, citing age or their high physical demand as reasons.

### Surgery

Surgical procedures were performed using a limited medial midvastus approach or lateral parapatellar approach, depending on the compartment involved. For tibio-femoral UKA, two different designs of implants were used: Journey II Uni (Smith and Nephew^®^, Andover UK, cutting type for the femur and a fixed bearing polyethylene) in 26 cases and HLS Uni evolution (Tornier®, Saint-Ismier, France, resurfacing type prosthesis with a monoblock, all polyethylene tibial component) in 8 cases. Concerning PFJ implants, two designs of the implant were used: JOURNEY (Smith and Nephew^®^, Andover UK) in 8 cases and Kneetec PFJ (Tornier^®^, Saint-Ismier, France) in one case. All implants were cemented. Two senior surgeons performed all UKA included in this study. This institution is considered a high-volume center for UKA, performing approximately 100 UKA per year.

The BlueBelt Navio image-free robotic surgical system (Smith and Nephew^®^) [7, 8] was used for all UKA (medial or lateral UKA and PFA).

### Anterior cruciate ligament surgery

**Figure 2:**
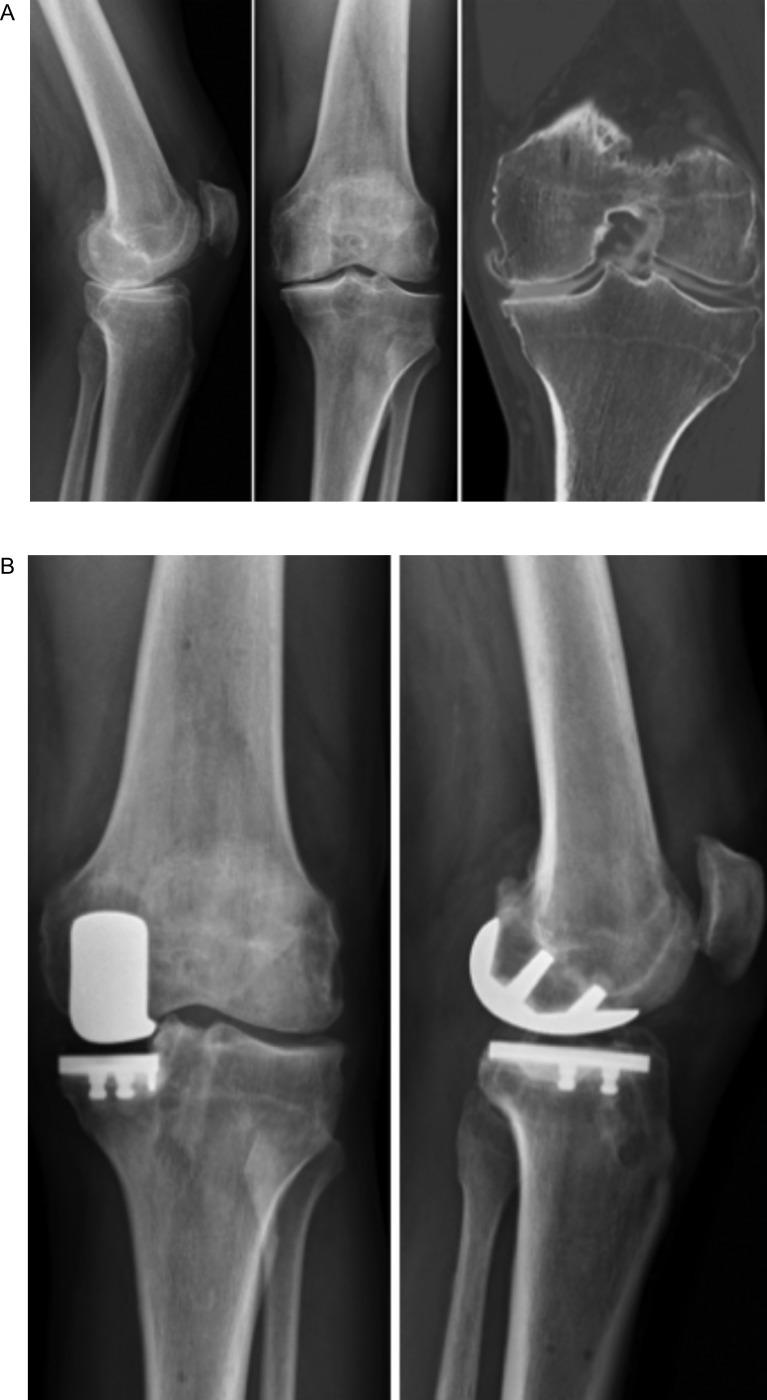
Patient of 68 years old with a prior history of medial meniscectomy, limduccited in his physical activities (cycling, highking) by medial tibiofemoral pain. The physical exam and the radiographic investigations found an anterior laxity (soft and-point to Lachman-Trillat test) and the lack of ACL with medial OA (A). An ACL reconstruction with medial UKA was performed (B). The patient was very satisfied at 18 months follow-up with no residual pain, no instability, and a complete return to sport.

Nine patients underwent a UKA after previously having ACL reconstruction. These patients had no anterior laxity clinically or complaint of knee instability (Figure 2). MRI confirmed the integrity of the graft. The UKA, in this case, is performed in a standard fashion with the usual care to protect the ACL.

Eleven patients underwent combined ACL reconstruction with UKA. Hamstring graft with interference screw fixation was used for all ACL reconstruction’s using an outside-in drilling technique. Briefly, the graft is harvested (gracillis and semitendinosus) while maintaining its distal insertion. The femoral tunnel is drilled first using an outside-in guide centered on the footprint of the native ACL attachment, and the tibial tunnel is positioned in the anterior part of the ACL footprint. Prior to graft passage and fixation, the robotic UKA procedure is performed, and cementation of the definitive implants is performed. Finally, the ACL graft is passed using shuttling sutures and fixation performed with two absorbable interferences screws. Postoperative rehabilitation is similar to classical rehabilitation after UKA.

### High tibial osteotomy

**Figure 3:**
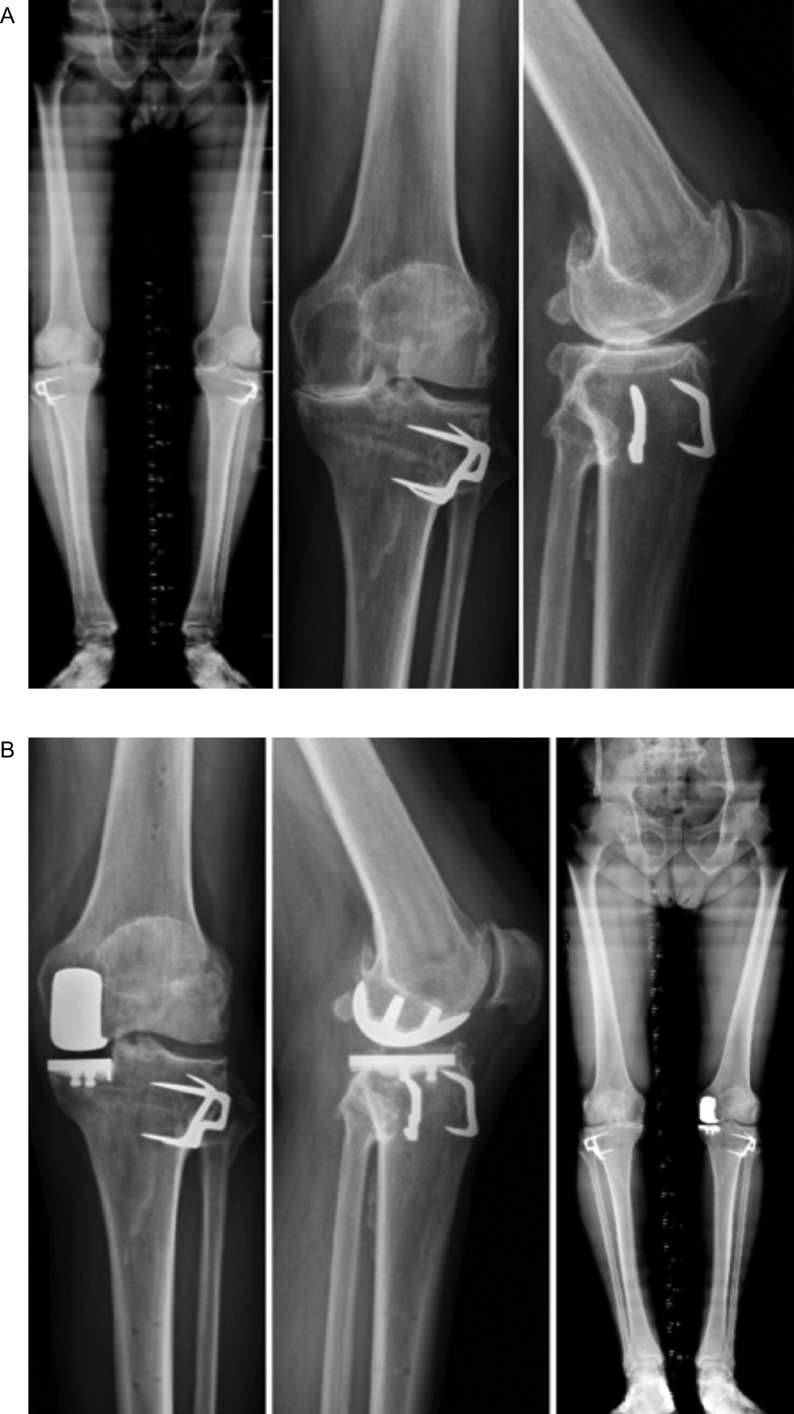
Active patient of 57 years old with a prior history of closing wedge high tibial osteotomy 14 years ago. He progressed medial OA with a varus deformity of 5° completely reducible (A). A medial UKA was performed to treat the OA and compensate for the intra-articular wear and the varus deformity (B).

In 3 cases, HTO was performed before UKA, 13.7 years ago ± 6.5 (7–20). In all cases, there was a progression of medial OA and varus deformity (Figure 3). In 1 case, a medial closing HTO was performed concomitantly with a UKA to correct a valgus deformity that was iatrogenically created previously by a medial opening HTO.

The medial closing HTO was performed under fluoroscopic control using a biplanar osteotomy. The target was to re-establish constitutional alignment, typically a hip- knee- Ankle angle (HKA) of 175 and the osteotomy fixed with 2 staples. Following this, UKA is performed with robotic assistance, and the remaining varus alignment is completely corrected with the prosthesis to end with a final HKA of 178–180°. During 2 cases, an ACL reconstruction was also performed to treat an associated anterior laxity. In those two cases, the first step consists of the ACL procedure with graft harvesting and tunnel drilled. Second, the HTO is performed and fixed as described, and finally, the UKA procedure and graft fixation is performed.

### Bicompartmental procedure

**Figure 4:**
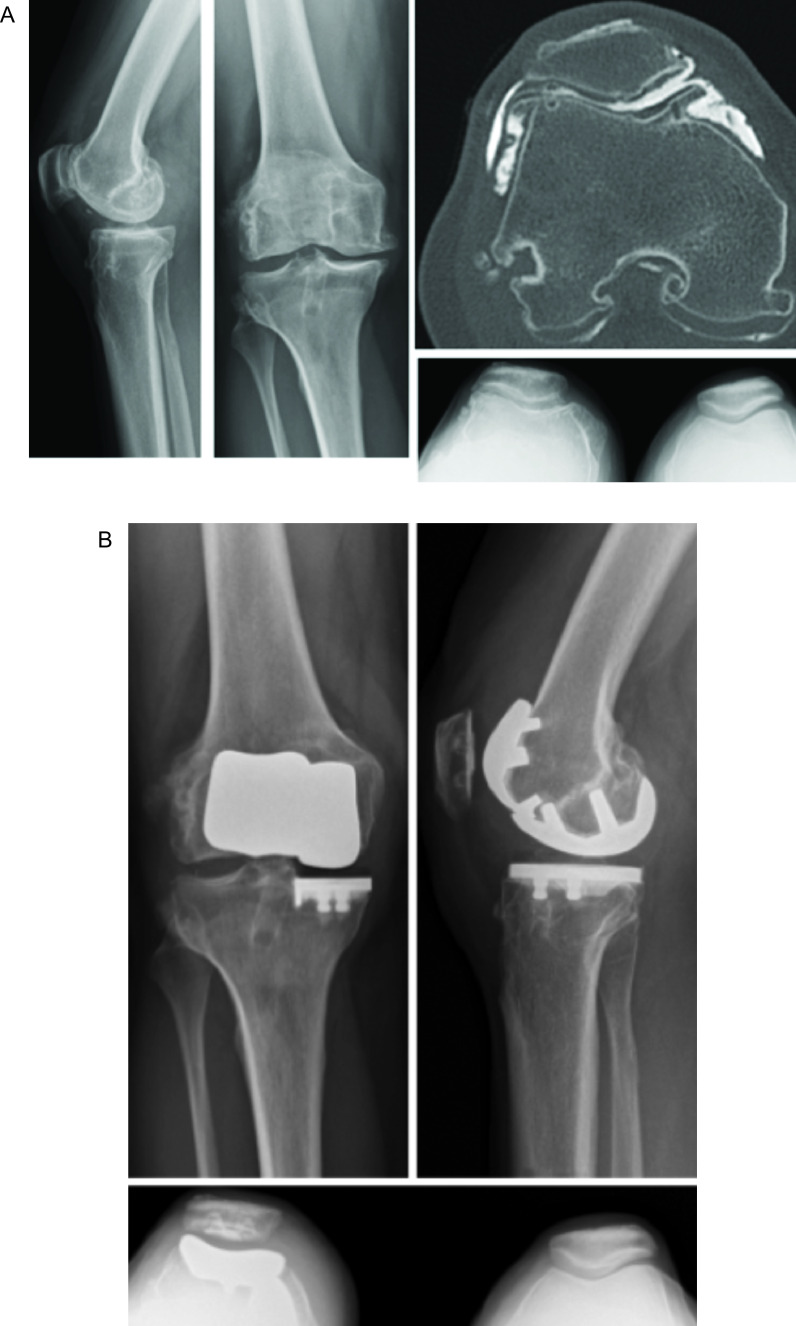
Woman of 48 years old with a prior history of ACL reconstruction 30 years ago. She is an active patient and is now limited by medial tibiofemoral, and patellofemoral osteoarthritis confirmed on X-rays and Arthro-CT scan (A). Physical examination found no anterior laxity and no pain in the lateral compartment. Patellofemoral and medial unicompartmental knee arthroplasty were performed simultaneously with robotic assistance (B). The patient was very satisfied at 2 years follow-up with no residual pain and returned to a normal life.

Six patients underwent PFA with UKA (5 medial UKA and 1 lateral UKA) with robotic assistance. Patients were identified as having symptomatic bicompartmental OA confirmed with either an Arthro-CT scan (Figure 4) or MRI scan.

**Figure 5:**
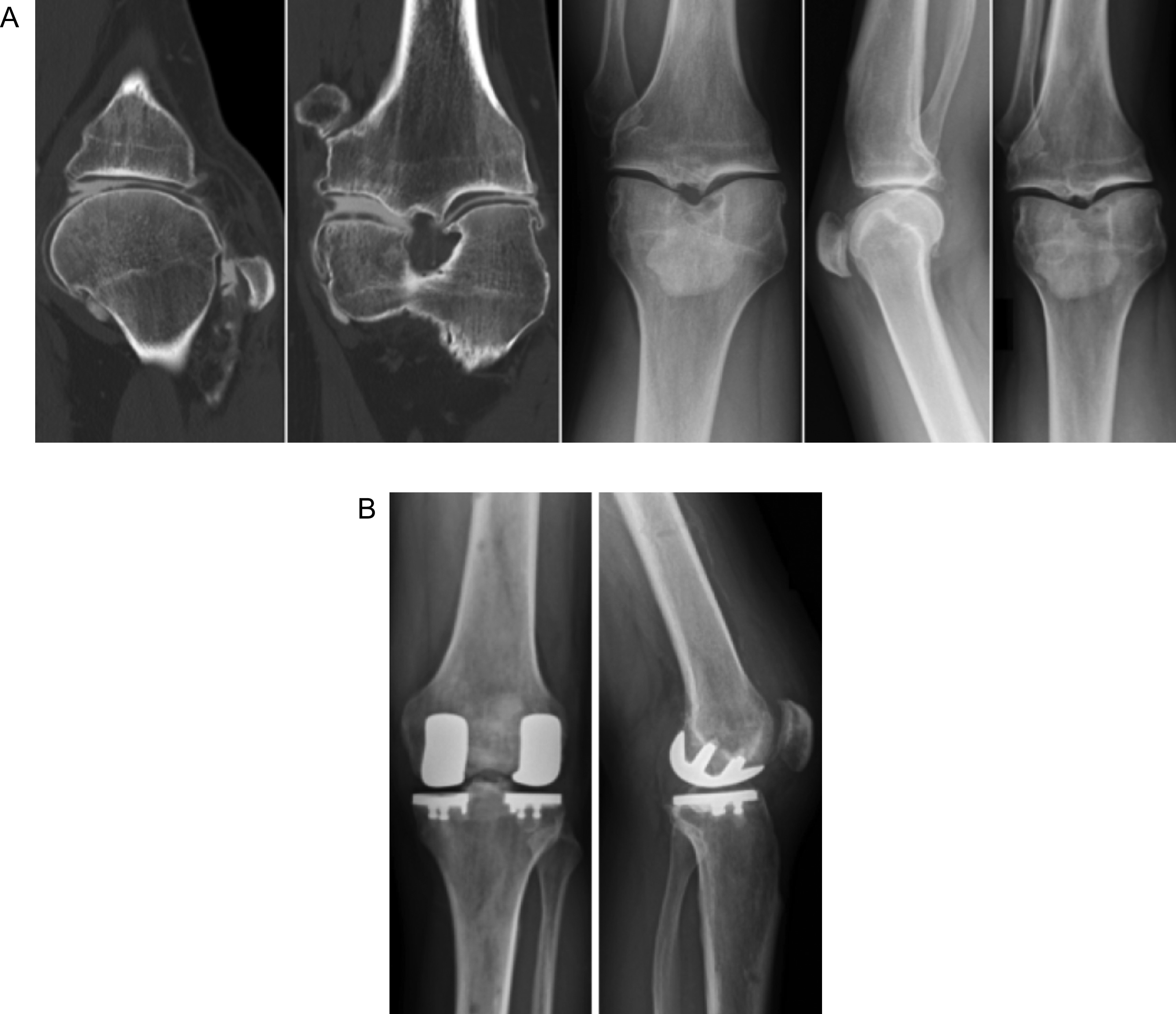
patient of 43 years old with a prior history of complete medial and lateral meniscectomy. X-rays and Arthro-CT scan confirm the medial and lateral OA with an evaluated chondral lesion (A). A Bi-compartmental procedure was performed with robotic assistance (B).

One patient underwent a bi-UKA (medial and lateral UKA, Figure 5), and one patient underwent a tri-UKA (medial, lateral, and PFA UKA). An Arthro-CT scan confirmed the localization of the chondral lesions, and the integrity of cruciate ligaments was confirmed both during pre-operative assessment and intra-operatively. In both cases, the patients were considered young (40 and 36 years old), had undergone previous meniscectomy, and participated in sports (running, soccer, hiking). Surgery for both cases was performed through an anterior skin incision with two separate arthrotomies. The first step was to correct any deformity with the UKA, i.e. In the case of valgus deformity, to start with the lateral UKA and conversely for a varus deformity with a medial UKA. A medial mid-vastus approach was performed for the medial UKA (and the PFJ) and a lateral mid-vastus approach for lateral UKA.

### Evaluation

Clinical evaluation score and patient satisfaction were collected at the last follow-up using the Knee Society Score (KSS) knee and function scores [9] and the Forgotten Joint Score (FJS) [10–12]. Radiological analysis (standard antero-posterior and lateral knee radiographs, patellar axial view radiograph, and full-length bilateral standing radiograph) was performed at the last follow-up. The complication rate was evaluated at the last follow-up, including all indications of reintervention and all reintervention procedures.

### Ethics approval

All procedures performed in studies involving human participants were in accordance with the ethical standards of the institutional and/or national research committee and with the 1964 Helsinki declaration and its later amendments or comparable ethical standards. For this type of study, formal consent was not required.

### Statistical analysis

Statistical analysis was performed with the online software EasyMedStat^®^ (http://www.easymedstat.com; Neuilly-Sur-Seine; France). The distribution of continuous variables were reported as mean with range and standard deviation. Statistical analysis was performed using the Student t-test or Wilcoxon nonparametric test. Categorical variables were compared using a Fisher exact test. Survival analysis was conducted with reintervention as the endpoint. Global survival curves were estimated with the Kaplan-Meier model, and the comparison of survivorship between the different initial etiologies was estimated with log-rank. The level of significance was set at *p* < 0.05 for all tests.

## Results

The population of the study was divided in 3 groups: UKA with ACL surgery, UKA with HTO, and Bicompartmental procedures (Figure 1). Results for each group individually are described in Tables 1 and 2.

**Table 1:** Patient demographics depending on the group.

	UKA and anterior laxity (*n* = 20)^a^	UKA and HTO (*n* = 4)^a^	Bicompartmental procedure (*n* = 8)^a^	*p* value
Age at surgery (year)	58 ± 6.7 (47–71)	64 ± 21 (33–84)	48 ± 6.7 (38–57)	0.03
Follow-up (months)	30 ± 17 (7.3–61.1)	20 ± 5 (12.6–27.1)	18 ± 5 (8–24)	0.04
Sex (female)	13 (65%)	0	6 (75%)	0.04
BMI	26.5 ± 3.8 (19–33)	26 ± 4.7 (19–32)	25 ± 4.8 (19–35)	n.s.
ASA score				
ASA 1	12	1	6	
ASA 2	8	1	2	
ASA 3	0	2	0	
Preoperative flexion ROM (°)	130 ± 8 (110–150)	130 ± 6 (130–140)	129 ± 7 (120–140)	n.s.
Preoperative mFTA (°)	178 ± 6 (168–192)	179 ± 6 (172–186)	177 ± 3 (174–183)	n.s.

a Data are presented as mean ± standard deviation [minimum–maximum] or number (proportion).

BMI: body mass index (kg/m^2^); ASA: American Society of Anesthesiologists; mFTA: mechanical femorotibial angle; ROM: range of motion; n.s.: non-significant.

**Table 2:** Comparison KSS scores, FJS, complication requiring new surgery and revision by groups and for the global population.

	UKA and anterior laxity (*n* = 20)^a^	UKA and HTO (*n* = 4)^a^	Bicompartmental procedure (*n* = 8)^a^	Global population (*n* = 32)^a^	*p* value
Preoperative KSS					
Knee	60.2 ± 11 [30–80]	44 ± 11.9 (30–59)	65.1 ± 9.2 [56–86]	59.8 ± 12.4 [30–86]	n.s.
Function	64.2 ± 15 [25–90]	53 ± 12.4 (35–69)	56.3 ± 16.5 [30–90]	60.8 ± 16 [25–90]	n.s.
Total	124 ± 21 [73–170]	97 ± 17 (70–117)	121.4 ± 13.7 [107–146]	120.2 ± 21.3 [70–170]	n.s.
Postoperative KSS					
Knee	93 ± 7.2 [79–100]	97.8 ± 2 (94–99)	89.8 ± 10.9 [69–100]	92.9 ± 8 [69–100]	n.s.
Function	93 ± 13.6 [60–100]	100 ± 0 (100–100)	92 ± 9.8 [80–100]	93.8 ± 12.1 [60–100]	n.s.
Total	184 ± 20 [139–200]	198 ± 2 (194–199)	181.8 ± 19.5 [149–200]	186 ± 18.9 [139–200]	n.s.
KSS improvement					
Knee	32.9 ± 14.5 [12–64]	54 ± 13.6 (35–69)	22.8 ± 9 [12–35]		n.s.
Function	29 ± 17 [0–75]	47 ± 12.4 (31–65)	42 ± 20.4 [10–70]		n.s.
Total	60.5 ± 28 [24–127]	101 ± 19 (77–129)	64.8 ± 22.9 [32–85]		n.s.
Postoperative FJS	78 ± 20.5 [40–100]	76.5 ± 11 (62–88)	73.7 ± 20.8 [40–100]	76.1 ± 19.6 [40–100]	n.s.
Postoperative complications	3 (15%)	0	4 (50%)	0	
Revision implant	1 (5%)	0	1 (12.5%)	0	

a Data are presented as mean ± standard deviation [minimum–maximum] or number (proportion), n.s.: non-significant.

### Demographics

Thirty-two patients (13 men and 19 women) were included. The mean age was 56.2 years ± 11.1 (range, 33–84) with a mean follow-up of 26.3 months ± 15 (7.3–61.1) and a mean BMI of 26.2 ± 4.2 (19–35). Patients undergoing bicompartmental arthroplasty were significantly younger than the HTO and ACL combined surgery groups (48 vs. 64 and 58 respectively, *p* = 0.03). For all other baseline demographics, there were no significant differences between groups.

### Clinical outcomes

In all groups there was a significant improvement between the pre- and postoperative KSS Knee (+34.3 ± 16.5 [12–69]), Function (+34.3 ± 18.6 [0–75]) and Total scores (+68.5 ± 29.4 [24–129]) (*p* = 0.001 – Table 2).

Patient satisfaction scores taken at last follow-up (*n* = 30) were satisfied (3; 10%) or very satisfied (27; 90%). The mean flexion at the last follow-up was 122° ± 6 (120–140).

### Survival

The implant survival rate was 94% at the last follow-up. Two patients (6.2%) underwent revision to TKA. The first patient (UKA and PFA) had revision for pain and stiffness after a failed arthroscopic arthrolysis. The second patient (UKA with ACL) presented with pain and was found to have aseptic loosening of the tibial component at 5 years follow-up with no intercurrent surgery.

### Complications

Seven patients (21.8%) experienced persistent stiffness with limited flexion under 90° requiring an arthroscopic arthrolysis. The mean time between UKA and arthrolysis was 6.8 months ± 9.6 (1–26.4). Of the UKA patients experiencing stiffness, 3 had associated ACL surgery and were 4 bicompartmental procedures (3 UKA + PFA and 1 Bi-UKA). All these patients, except one, recovered their range of motion. No difference was observed for KSS and FJS scores, as well as complications between groups (Table 3). No intraoperative complications or infections were observed at the last follow-up.

**Table 3:** Comparison of KSS and satisfaction between patient presenting a postoperative complication and patient with no complication.

	No postoperative complication (*n* = 24)^a^	Postoperative complication (*n* = 7)^a^	*p* value
Preoperative KSS			
Knee	60.7 ± 12.5 [30–86]	53.7 ± 11.2 [35–70]	n.s.
Function	59.5 ± 16.6 [25–90]	63.9 ± 14.2 [50–90]	n.s.
Total	120.2 ± 22.9 [70–170]	117.6 ± 14.4 [100–146]	n.s.
Postoperative KSS			
Knee	94.4 ± 5.7 [79–100]	82.7 ± 12.4 [69–99]	n.s.
Function	93.6 ± 12.6 [60–100]	95 ± 8.7 [80–100]	n.s.
Total	187.5 ± 18 [139–200]	176 ± 20.6 [149–199]	n.s.
KSS Improvement			
Knee	33.6 ± 15.6 [12–69]	38.7 ± 21.3 [12–64]	n.s.
Function	34.5 ± 19.9 [0–75]	33.3 ± 9 [20–43]	n.s.
Total	68.5 ± 30.6 [24–129]	68.7 ± 18.9 [42–84]	n.s.
Postoperative FJS	78.2 ± 19.2 [40–100]	66 ± 17.9 [40–91]	n.s.

a Data are presented as mean ± standard deviation [minimum–maximum] or number (proportion), n.s.: non-significant.

KSS = Knee society score; FJS = Forgotten Joint Score.

Concerning stiffness, arthroscopic arthrolysis was performed after a mean delay of 0.57 months ± 0.8 (0.08–2.2) for flexion at 69° ± 19 (40–90).

## Discussion

The most important finding of this study was the good final clinical results despite reintervention for stiffness being required in a high proportion of cases (21.8%). UKA with additional procedures is a promising solution to the physically active patient with monocompartmental knee arthritis in the presence of associated relative contraindications of an ACL deficient knee, coronal plane deformity, or bi-compartmental disease with good clinical outcomes at short term follow-up comparable to isolated UKA [13].

The surgical management of active patients with symptomatic full-thickness chondropathy and associated pathology such as extra-articular deformity, defunctioned meniscus, or anterior cruciate ligament remains a significant challenge. UKA has many benefits in such a population, however it’s use is restricted by a significant number of contraindications, and concern remains regarding the outcomes of performing UKA with associated surgery to deal with the additional pathology. In appropriately selected patients who have been fully counseled on the management options, we demonstrated UKA with associated procedures has good short-term survivorship and PROM’s. Patient communication in this setting is critical, and it is important to note that in the current study, all patients had been offered TKA and declined. While the results are promising, these results are short-term, and we did observe a high rate of stiffness, particularly in those undergoing associated ACL surgery, and this will inform our rehabilitation protocols in the future.

Performing UKA in ACL-deficient knees appears to be a relative contra-indication. A recent study by Kikuchi et al. [14] reported good outcomes combining UKA and ACL reconstruction in anteromedial OA. Several additional studies have demonstrated restoration of normal knee kinematics and similar postoperative knee score to our results [15, 16]. Pandit *et al.* [15] found excellent results in a cohort of 15 patients with combined ACL reconstruction and medial UKA (Oxford design) with no difference with a matched compared population of UKA with intact ACL. They reported high knee scores (KSS > 95) similar to our results.

UKA in the presence of chondropathy in an additional compartment continues to be a problematic scenario for orthopedic surgeons. A small number of studies have examined combined tibio-femoral UKA with PFJ arthroplasty. Romagnoli *et al.* [17] and Benazzo *et al.* [18] report good results with improved knee joint range of motion, clinical and functional score, and excellent survivorship similar to isolated PFA. These results are similar to Rossi *et al.* [19], who reported in a series of 57 combined procedures (PFA associated with a medial or lateral UKA) excellent clinical and radiological outcomes with a good survival rate at mid-and long-term follow-up. Confalonieri *et al.* [20], in a matched paired study, suggested that Bi-UKA (medial and lateral UKA) is a viable option for bicompartmental tibio-femoral OA as well as TKA with a higher level of function and no over-risk of reintervention. Paratte *et al.* [21] reported 17-year implant survival to revision, radiographic loosening, or disease progression 78% in a bi-UKA group and 54% in a medial UKA + PFA group. With the development of prosthetic design and surgical tools such as robotic platforms, the long-term follow-up of studies such as the present one will inform clinical management decisions with increasing confidence.

We observed a high rate of postoperative stiffness requiring arthroscopic arthrolysis. This high rate could be explained by the complexity of cases. The correction of all these anomalies will impact the biomechanics of the knee and impact the rehabilitations. Fournier *et al*. [22] reported a high rate of stiffness after isolated UKA with a good result of arthroscopic arthrolysis to treat this complication similar to our results. Each procedure (ACL reconstruction, HTO, UKA, PFP) could potentially be complicated with postoperative stiffness. The addition of several procedures seems to increase the global risk of postoperative stiffness. Concerning stiffness, we preferred to perform arthroscopic arthrolysis to treat the stiffness firstly to avoid any chondral lesion on the patellofemoral joint due to hyper pressure during the manipulation in flexion. We performed arthroscopy to remove all the intra-articular adhesions and, after, to force progressively the knee in flexion. We thought that manipulation under anesthesia could be traumatic for the patellofemoral cartilage and the ligament because in case of stiffness, all the tissues are retracted, and the forced flexion could create some iatrogenic stretching. All the patients benefited from fast postoperative rehabilitation protocol with full weight-bearing, rehabilitation protocol with knee flexion on the first day with continuous passive motion with Kinetec Spectra Essential™ (Kinetec^®^), and regular physical therapy. Multimodal pain protocol was applied for all patients with periarticular infiltration, peripheral nerve block, NSAIDs, and painkiller.

Concerning the old patients of this study, it concerned only two patients aged more than 75 years old. These patients were very active, practicing impact sport and refused categorically a TKA. They have a bad opinion concerning TKA thinking they could not practice their physical activities after this procedure.

This study has several limitations. Firstly, it is a retrospective analysis with a limited follow-up and a limited number of patients. Nevertheless, the indication of combined procedures is very limited, explaining the small cohort in the literature similar to our study. Secondly, the population is heterogeneous with several combined procedures on the same knee. The results thus should be interpreted with caution and the results not generalized. The aim of this study was to describe our indication, surgical techniques, and results at short-term follow-up. The implications of this study are that for selected cases for which a TKA may be recommended but refused by the patient, UKA with an associated procedure to address the contraindication is possible, and the early results are promising. Both the patient and surgeon must be aware of the high rate of reintervention for stiffness, however, with optimized rehabilitation protocols, outcomes and patient satisfaction are promising.

## Conclusion

This study suggests that performing UKA with an additional procedure to address relative contraindication’s to the arthroplasty in physically active patients with monocompartmental knee arthritis is an efficient strategy with good results at short-term follow-up. It should be reserved for patients where TKA is likely to have unsatisfactory results, and the patient has been fully counseled regarding the management options. Even if there is a high rate of stiffness requiring a re-intervention, the final results are very satisfying with no impact of the reintervention on the clinical result in the short term.

## Data availability

Availability of data and materials was respected and preserved for this study.

## Conflict of interest

No benefits in any form have been received or will be received from a commercial party related directly or indirectly to the subject of this article.

## Funding

There is no funding source.

## Ethical approval

Ethical approval was not required.

## Informed consent

Consent to Participate was respected for this study.

Consent to Publish was respected for this study.

## Authors contributions

V. Derreveaux: Writting original draft.

A. Schmidt: Writting, Reviewing.

J. Shatrov: Writting, Reviewing.

E. Sappey-Marinier: Supervision.

C. Batailler: Supervision.

E. Servien: Supervision.

S. Lustig: Supervision, Conceptualization, Methodology.

## Level of evidence

IV: retrospective or historical series.
